# Impact of Diabetes Mellitus on the Risk of End-Stage Renal Disease in Patients with Systemic Lupus Erythematosus

**DOI:** 10.1038/s41598-018-24529-2

**Published:** 2018-04-16

**Authors:** Ming-Yan Jiang, Jyh-Chang Hwang, I-Jung Feng

**Affiliations:** 1 Division of Nephrology, Chi Mei Medical Center, Tainan, Taiwan; 2 Department of Hospital and Health Care Administration, Chia Nan University of Pharmacy and Science, Tainan, Taiwan; 3 Division of Medical Research, Chi Mei Medical Center, Tainan, Taiwan

## Abstract

Systemic lupus erythematosus (SLE) patients are associated with insulin resistance and are at higher risk to develop diabetes mellitus (DM). SLE and DM **could** lead to renal failure respectively. However, it is unknown whether DM increases the risk of end-stage renal disease (ESRD) in SLE patients. This study aimed to evaluate potential synergistic effect of DM on SLE patients for development of ESRD. We conducted this study by using National Health Insurance Research Database of Taiwan. We recruited SLE patients with newly-diagnosed DM as the study cohort. A comparison cohort at a 1:1 ratio of SLE patients without DM matched by age, sex, age at the diagnosis of SLE, duration between diagnosis of SLE and DM, and various comorbidities through propensity score matching were recruited. After 5.01 ± 3.13 years follow-up, the incidence of ESRD was significantly higher in the DM group than in the non-DM group (Incidence rate ratio: 2.71; 95% CI: 1.70–4.32). After control of confounding factors, DM was not an independent risk factor of ESRD. After starting dialysis, DM patients had a similar mortality rate to those without DM. **In summary**, **SLE patients superimposed with subsequent DM are associated with potentially higher risk to develop ESRD**.

## Introduction

Systemic lupus erythematosus (SLE) is a multi-organ involved autoimmune disease. Renal involvement is one of its most severe manifestations and is a major cause of morbidity and mortality^1^. The risk for end-stage renal disease (ESRD) was significantly higher in the patients with SLE than in the non-SLE population^2^. Up to 60% of lupus patients have varying degrees of renal involvement. Around 10% to 30% of patients with severe lupus nephritis progresses to ESRD within 15 years of diagnosis^3^. In a retrospective study in Taiwan, 2.5% of newly diagnosed SLE patients developed ESRD after 6-year follow-up^4^. In developed countries, the 5-, 10-, and 15-year incidences of ESRD in patients with lupus nephritis were around 11%, 17% and 22%, respectively^5^.

SLE is also associated with an increased risk of development of diabetes mellitus (DM). Patients with SLE had tendencies to develop other autoimmune disease, such as type 1 diabetes^6^. Besides, a cohort study in Toronto documented that women with SLE had a significantly higher prevalence of diabetes mellitus than the age-matched healthy controls (5% versus 1%)^7^. Lupus patients had a higher level of insulin resistance and hyperinsulinemia than age-matched healthy controls^8^. Anti-insulin antibodies^9^ and chronic inflammation^10^ were associated with hyperinsulinemia and insulin resistance, and may account for the development of DM in SLE patients. In addition, corticosteroid, a mainstream medication for SLE, is also associated with significantly higher levels of blood glucose^11^. In a Malaysian cohort, the prevalence of DM among SLE patients treated with prednisolone was 13%^12^. Therefore, patients may tend to develop DM after acquiring SLE.

SLE patients have increased risks for developing DM. On the other hand, DM is often accompanied with diabetic nephropathy^13^, which is the leading cause of ESRD^14,15^. DM and SLE lead to renal failure in different pathways and are two independent risk factors for ESRD. However, it is unclear whether DM has an additive effect on SLE patients in the progression to ESRD. Is DM an independent factor to aggravate renal function deterioration in SLE patients? Furthermore, diabetic dialysis patients have worse long-term survival than that of non-diabetic patients^16^. In contrast, the 5-year survival of incident ESRD among SLE patients are similar to that of non-SLE age- and sex-matched controls^4^. The difference of the long-term survival between patients with SLE alone and SLE concomitant with DM after development of ESRD is still unknown.

In this study, we investigated the potential additive effects of DM on SLE patients to ESRD by using population-based data in Taiwan to compare the incidence rates of ESRD in SLE patients with and without DM. We also compared the long-term survival of these two groups of patients to examine the potential additive effect of DM on lupus ESRD patients’ survivals. We hypothesized that patients with both SLE and DM were at higher risk for developing ESRD and had worse long-term survival.

## Materials and Methods

### Data Source

We conducted a nationwide survey of lupus patients in Taiwan by using the National Health Insurance Research Database (NHIRD). The NHIRDs of Taiwan contains a large sample size and high validity of disease diagnosis. The National Health Research Institute managed and maintained all insurance claim data for research purpose, and information of each individual patient in the NHIRDs were encrypted and de-identified. Therefore, informed consent was waived. The Research Ethics Committee of the Chi Mei Medical Center approved this study. All the methods in this research were performed in accordance with the relevant guidelines and regulations.

### Study design and Study population

The inclusion criteria of the subjects in this study were those with a first diagnosis of SLE according to the International Classification of Disease, Revision 9, Clinical Modification (ICD-9- CM) code 710.0 from January 1, 2000 to December 31, 2011. We excluded the patients who had DM prior to the diagnosis of SLE. We defined the date of diagnosis of DM as the index date. Lupus patients with newly-diagnosed DM were selected as the study cohort (DM group, n = 1317). By means of propensity score, a 1:1 ratio of non-diabetic incident SLE patients with matched sex, age at SLE diagnosed (±30 days), duration between SLE to index date and selected comorbidities by propensity score matching were recruited as comparison cohort (non-DM group, n = 1317). The index date in the comparison cohort was created by matching the year of the case’s index date. The definition of DM is based on either two of the following conditions: (1) at least 1 time of diagnosis of DM, or a prescription for anti-diabetic medication (ICD9 = 250, A181, A189, A229, A239, 3572, 3620) in inpatient claim data; (2) at least 2 times of diagnoses of DM, or at least 1 time of diagnosis of DM with a prescription for anti-diabetic medication in ambulatory claim data. The primary outcome was the development of ESRD by tracing subjects’ medical records until December 31, 2012. Patients were diagnosed as ESRD if they received maintenance dialysis for more than 90 days. ESRD were reconfirmed by receiving a catastrophic illness certificate (CIC) with the ICD-9-CM (International Classification of Disease, Revision 9, Clinical Modification) code 585. We also compared the long-term survival in SLE patients with and without DM. Subjects were followed up to the date of death or December 31, 2012. We linked to inpatients claim data to find if these recorded the expiry date in CIC or lost NHI coverage for more than 30 days.

### Definition of co-morbid conditions

By using ambulatory and inpatient claim data with three or more outpatient visits or at least one hospitalization record, we searched the database to determine if subjects had hypertension (ICD code no. 401 to 405), coronary artery disease (CAD) (ICD code no. 410 to 414), hyperlipidemia (ICD code no. 272), gout (ICD code no. 274), hepatitis B virus (HBV) infections (code no. 07020 to 07022, and 07030 to 07032), hepatitis C virus (HCV) infections (code no. 07041 to 07044 and 07051 to 07054) and liver cirrhosis (code no. 465.2).

### Statistical Analysis

The Student t test and *X*^2^ test were used for comparisons of baseline continuous and categorical variables, respectively, between the lupus patients with and without DM. The risk of getting ESRD was compared between the DM and non-DM group by estimating incidence rate ratio (IRR) with Poisson regression. Cox proportional hazard analysis was further performed to analyze the risk factors for developing ESRD during the follow-up period. The cumulative incidence rates for developing ESRD and the survival rates of the 2 groups were determined by the Kaplan–Meier method. A log rank test was applied to compare the difference between 2 survival curves after the development of ESRD for the patients on maintenance dialysis. A P-value of less than 0.05 was considered to be statistically significant. All of the analyses were conducted using SAS statistical software (version 9.3.1, SAS Institute, Cary, NC).

### Data availability statement

All data used for this project are obtained from the National Health Insurance Research Database (NHIRD) published by Taiwan National Health Research Institutes (NHRI). The NHRI is a nonprofit foundation established by the government. The use of NHIRD is limited to research purposes only. Only citizens of Taiwan who fulfill the requirements of conducting research projects are eligible to apply for the NHIRD. Due to legal restrictions imposed by the government of Taiwan in relation to the “Personal Information Protection Act”, data cannot be made publicly available. Requests for data can be sent as a formal proposal to the NHIRD (http://nhird.nhri.org.tw). Applicants must follow the “Personal Information Protection Act” and related regulations of National Health Insurance Administration and NHRI, and an agreement must be signed by the applicant and his/her supervisor upon application submission.

### Ethical issues

The research ethics committee of the Chi Mei Medical Center has approved this study.

## Results

The mean age at SLE diagnosis was 44.56 years in this cohort. The mean age at DM diagnosis was 48.5 years and the mean duration from SLE diagnosis to the date of DM diagnosis was 3.97 years. Most of our study subjects were female (91%). Compared to non-DM counterparts, SLE patients with DM had higher prevalence rates of co-morbidities, including hypertension (31% vs. 4%), CAD (6.3% vs. 1.1%), hyperlipidemia (10.6% vs. 1.1%), gout (3.1% vs. 0.4%), HBV infection (1.21% vs. 0.30%), HCV infection (1.7% vs. 0.2%), and liver cirrhosis (2.0% vs. 0.2%) (Table 1).

**Table 1:** Baseline Demographic Characteristics of SLE patients with and without DM.

	DM (N = 1317)	Non-DM (N = 1317)	P-value
Age at SLE, years (mean ± SD)	44.56 ± 15.06	44.56 ± 15.06	0.99
Age at index date, years (mean ± SD)	48.52 ± 14.99	48.52 ± 14.99	0.99
Age at index date (n, %)			>0.99
<45	541 (41.08)	541 (41.08)	
45–65	576 (43.74)	576 (43.74)	
≥65	200 (15.19)	200 (15.19)	
Duration from SLE date to index date, years (mean ± SD)	3.97 ± 2.93	3.97 ± 2.93	>0.99
Female (n, %)	1198 (90.96)	1198 (90.96)	>0.99
Baseline comorbidity (n, %)			
Hypertension	412 (31.28)	55 (4.18)	<0.0001
CAD	83 (6.30)	14 (1.06)	<0.0001
Hyperlipidemia	140 (10.63)	14 (1.06)	<0.0001
Gout	41 (3.11)	5 (0.38)	<0.0001
HBV infection	16 (1.21)	4 (0.30)	0.007
HCV infection	22 (1.67)	3 (0.23)	0.0001
Cirrhosis	26 (1.97)	3 (0.23)	0.001

SLE: systemic lupus erythematosus; CAD: coronary artery disease; HBV infection: hepatitis B virus infection; HCV infection: hepatitis C virus infection; SD: standard deviation.

Between DM patients and controls cohorts, the differences in categorical variables were compared using Chi-square tests or fisher’s exact test and the differences in continuous variables were compared using Student’s t-test.

**Figure 1:**
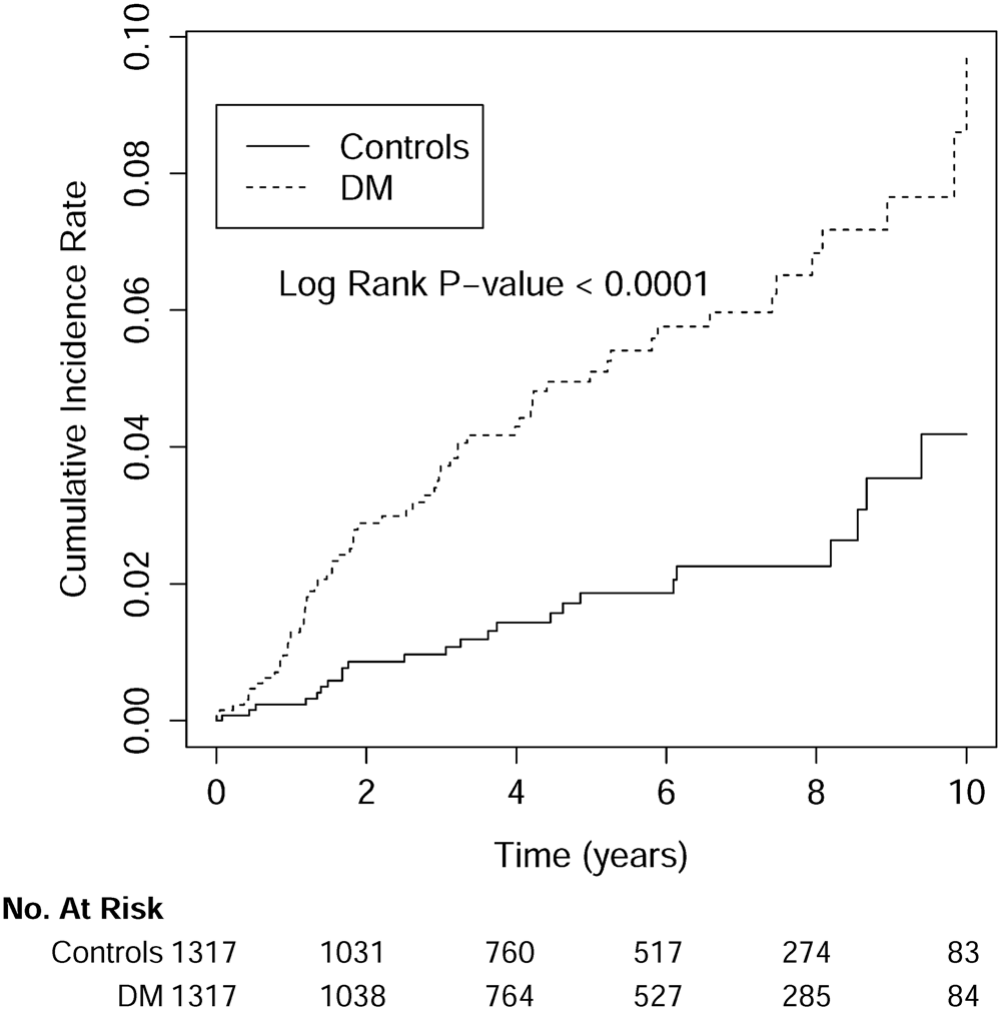
The cumulative incidence rate for developing end-stage renal disease was significantly higher in SLE patients with DM compared with those without DM (control group). The time zero was the index date, which was defined as the date of DM diagnosis in the study cohort (DM group) and the date created by matching the year of the case’s index date in the control group.

The mean follow-up period was 5.01 ± 3.13 years. As shown in Fig. 1, the cumulative incidence rate for developing ESRD was significantly higher in DM group compared to non-DM group (p < 0.0001). The cumulative incidence of ESRD was significantly higher in DM group than non-DM group [9.80 vs. 3.62 per 1000 person-years, p < 0.0001; incidence rate ratio (IRR): 2.71, 95% confidence interval (CI): 1.70–4.32)] (Table 2). The cumulative incidence of ESRD is more than twice higher in the DM group than in non-DM group in all age groups, especially in those with age older than 65 years. Both male (IRR: 4.76; 95% CI: 1.03–22.05) and female (IRR: 2.53; 95% CI: 1.55–4.14) lupus patients with DM had higher cumulative incidence of ESRD compared with the controls (Table 2).

**Table 2:** Subgroup Analysis of the Risk of ESRD by Age, Sex, Comorbidity and Follow-Up Duration for SLE patients with and without DM.

**Characteristics**	**DM**				**Non-DM**				**IRR (95% CI)**	***P*** **-value**
	**N**	**ESRD**	**PY** ^**#**^	**Rate** ^**a**^	**N**	**ESRD**	**PY** ^**#**^	**Rate** ^**a**^		
All	1317	65	6635.70	9.80	1317	24	6633.60	3.62	2.71 (1.70–4.32)*	<0.0001*
Age at index date										
<45	541	27	2851.67	9.47	541	12	2899.00	4.14	2.29 (1.16–4.51)	0.02*
45–65	576	25	2735.61	9.14	576	9	2698.15	3.34	2.94 (1.28–5.87)	0.01*
≥ 65	200	13	1048.42	12.40	200	3	1036.46	2.89	4.28 (1.22–15.03)	0.02*
Gender										
Male	119	9	575.02	15.65	119	2	608.64	3.29	4.76 (1.03–22.05)	0.04*
Female	1198	56	6060.68	9.24	1198	22	6024.96	3.65	2.53 (1.55–4.14)	0.0002*
Comorbidity										
Hypertension	412	36	1941.90	18.54	55	5	248.20	20.15	0.92 (0.36–2.35)	0.86
CAD	83	3	435.82	6.61	14	0	86.61	—	—	—
Hyperlipidemia	140	15	616.47	24.33	14	0	64.22	—	—	—
Gout	41	8	178.82	44.74	5	1	33.38	29.96	1.49 (0.19–11.94)	0.71
HBV	16	0	—	—	4	0	—	—	-	—
HCV	22	1	88.97	11.24	3	0	2.78	—	—	—
Cirrhosis	26	1	149.71	6.68	3	0	11.98	—	—	—
Follow-up period										
0–2 years	282	7	452.21	15.48	286	3	334.47	8.97	1.73 (0.45–6.67)	0.43
2–4 years	271	10	826.45	12.10	278	3	891.69	3.36	3.60 (0.99–13.07)	0.05
4–6 years	240	12	1174.73	10.22	245	4	1218.67	3.28	3.11 (1.00–9.65)*	0.04*
6–8 years	243	14	1660.44	8.43	241	3	1688.87	1.78	4.75 (1.36–16.52)	0.01*
≥ 8 years	281	22	2521.87	8.72	267	11	2499.90	4.40	1.98 (0.96–4.09)	0.06

^#^PY, person-years.

^a^Rate: per 1000 person-years.

IRR: Incidence rate ratio.

CI: Confidence interval.

ESRD: End stage renal disease; SLE: systemic lupus erythematosus; CAD: coronary artery disease; HBV infection: hepatitis B virus infection; HCV infection: hepatitis C virus infection.

The risk of getting ESRD was compared between the DM group and the controls group by estimating incidence rate ratio with Poisson regression.

Table 3 showed the crude and adjusted hazard ratios (HR) for development of ESRD in our study cohort. The crude hazard ratio among lupus patients with DM was significantly higher than those without DM (HR: 2.71; 95% CI: 1.70–4.33). However, after control of confounding factors, DM was not an independent risk factor of ESRD in lupus patients (adjusted HR: 1.64; 95% CI: 0.97–2.76).

**Table 3:** Crude and adjusted hazard ratios of Cox proportional hazard regressions and 95% confidence interval for ESRD during the follow-up period for study cohort.

Variable	Crude Hazard Ratio (95% CI)	Adjusted Hazard Ratio (95% CI)
DM (yes vs. no)	2.71 (1.70–4.33)*	1.64 (0.97–2.76)
Age at index date		
<45	1.00	1.00
45–65	0.92 (0.58–1.45)	0.71 (0.44–1.14)
≥65	1.13 (0.63–2.02)	0.74 (0.40–1.37)
Female (vs. male)	0.72 (0.38–1.35)	0.74 (0.40–1.40)
Comorbidity		
Hypertension (yes vs. no)	4.30 (2.84–6.53)*	3.29 (1.98–5.47)*
CAD (yes vs. no)	0.85 (0.27–2.68)	0.35 (0.11–1.16)
Hyperlipidemia (yes vs. no)	3.73 (2.14–6.51)*	1.77 (0.96–3.27)
Gout (yes vs. no)	6.90 (3.46–13.77)*	3.06 (1.46–6.42)*
HBV (yes vs. no)	N/A	N/A
HCV (yes vs. no)	0.64 (0.22–11.28)	1.22 (0.17–8.96)
Cirrhosis (yes vs. no)	0.00 (0.00 - Inf)	0.82 (0.11–6.01)

DM: diabetes mellitus; CAD: coronary artery disease; HBV infection: hepatitis B virus infection; HCV infection: hepatitis C virus infection.

*p-value < 0.05. CI: Confidence interval.

After entering dialysis, the DM group (n = 65) had a similar probability of survival rate compared with the non-DM group (n = 24) (Log Rank test p-value = 0.197). The risk of death among diabetic lupus patients who developed ESRD were not higher than those without DM (adjusted HR: 6.73; 95% CI: 0.74–61.25). However, the patient number was too low that we were unable to conclude about comparison of mortality rates of lupus ESRD patients with and without DM.

## Discussion

The main finding of this study was that SLE patients superimposed with DM had a higher cumulative incidence of ESRD than those without DM after long-term follow-up. Both male and female patients had increased cumulative incidence of ESRD after development of DM. However, the impact of DM on ESRD in patients with SLE attenuated after adjustment for age, gender, and comorbidities. Besides, the difference of long-term mortality rate between SLE patients with DM and the non-DM counterparts after entering dialysis remained undetermined.

Several studies had reported the co-existence of DM and SLE^17^. Bruce *et al*. reported that SLE patients had 5-fold higher risk to have DM^7^. Chronic inflammation and oxidative stress in SLE may contribute to insulin resistance^8^, which is a key component of DM^18^. SLE group had higher fasting insulin levels and homeostatic model assessment insulin resistance compared with healthy controls^19^. The association between insulin resistance and impaired glucose tolerance had been demonstrated in an animal model of SLE^20^. In addition, the medications for treatment of SLE such as glucocorticoid and mycophenalate mofetil (MMF) also tend to be diabetogenic^21^. The prevalence of DM among SLE patients treated with glucocorticoid had been reported as 13^12^ to 25%^22^. MMF also affect glycemic control, thus aggravating the impact of glucocorticoid therapy on the development of DM in SLE patients^22^. Because of insulin resistance and the adverse effects of immunosuppressive therapy, SLE patients are tended to develop DM.

Diabetic nephropathy is the leading cause of ESRD worldwide, accounting for approximate 40% of the population receiving renal replacement therapy^14,15^. Lupus nephritis is also an important cause of ESRD. In one study of 1008 SLE patients in the United States, 84 patients had DM concomitantly^23^. After a mean follow-up period of 4.8 years, they found that DM was not associated with elevated risk of ESRD^23^. Our finding suggested that DM was associated with increased cumulative incidence of ESRD in SLE patients. However, after adjusting for patients’ characteristics and comorbidities, DM is not an independent risk factor for ESRD in SLE patients. It seems that DM associated co-morbidity condition is an important risk factor for deteriorating renal function in lupus patients developed subsequent DM.

Lupus patients with DM may represent higher insulin resistance and more comorbidities, which predisposed them to develop ESRD. Insulin resistance had been found to be associated with kidney dysfunction, playing a role in glomerular hyperfiltration, endothelial dysfunction and increased vascular permeability^24^. A prospective cohort study demonstrated that insulin resistance was associated with a rapid decline in kidney function^25^. SLE patients had been found to be associated with increased risk of cardiovascular disease and metabolic syndrome^26^, which may be attributed to insulin resistance^8,27^. In our current study, we found that SLE patients with DM had more comorbid conditions such as hypertension, hyperlipidemia and gout, which are key components of metabolic syndrome. We hypothesized that SLE patients with DM may represent higher level of insulin resistance, which results in accelerated nephrosclerosis and worse renal outcomes.

To our knowledge, this is the first large-scale population-based study to establish the additive effect of DM on the development of ESRD in patients with SLE. Nonetheless, there are a few limitations. First, information about the histological type of lupus nephritis, response to treatment, and presence of antiphospholipid antibodies couldn’t be captured from our database. The disease activity of lupus and the medication for treatment were also not recorded. Higher SLE disease activity may cause more severe renal involvement. Some medications for treatment of SLE may also have various extents of nephrotoxicity, leading to renal function impairment. We matched the study and comparison cohorts to minimize these effects. Second, baseline renal function and the state of proteinuria were not evaluated. By using propensity score matching, we assumed that the baseline renal function of both groups was equal. Third, the details of blood glucose and blood pressure control were not available in the claim database, both of which are important risk factors related to an accelerated deterioration of renal function. Finally, we couldn’t find out that the diagnosis of DM in our study cohort were steroid-induced DM or type 2 DM. Steroid induced-DM may indicate more severe underlying SLE, as higher steroid is required to control the disease. This may also reflect a more severe histological type of lupus nephritis that causing progression to ESRD in our study subjects. However, we found that DM was not an independent risk for ESRD in patients with SLE after adjustment. This may indicate that, compared to DM per se, its related underlying conditions may have critical impact on SLE patients to develop ESRD.

In conclusion, SLE patients superimposed with DM was associated with higher cumulative incidence of ESRD. However, DM is not an independent risk factor for ESRD in SLE patients. The impact of DM on ESRD attenuated after adjusting for patients’ characteristics and comorbidities. It indicates that much more diabetes associated co-morbid conditions in SLE patients concomitant with succeeding DM play a role in aggravating renal function decline. Further studies are still needed to be conducted to clarify the association between DM and accelerating renal failure in patients with SLE. However, meticulous adjustment the dosage of steroid and immunosuppressants and careful monitoring patient’s glucose level are recommended on the management of the patients with SLE.
